# Tailoring Properties of Mixed-Component Oleogels: Wax and Monoglyceride Interactions Towards Flaxseed Oil Structuring

**DOI:** 10.3390/gels6010005

**Published:** 2020-01-31

**Authors:** Noadia G. Barroso, Paula K. Okuro, Ana P. B. Ribeiro, Rosiane L. Cunha

**Affiliations:** 1 Department of Food Engineering, Faculty of Food Engineering, University of Campinas, UNICAMP, Campinas CEP 13083-862, SP, Brazil; noadia_gb@hotmail.com (N.G.B.); paulaokuro@gmail.com (P.K.O.); 2 Department of Food Technology, Faculty of Food Engineering, University of Campinas, UNICAMP, Campinas CEP 13083-862, SP, Brazil; anabadan@unicamp.br

**Keywords:** soft matter, berry wax, sunflower wax, glycerol monostearate, organogel, fat replacer

## Abstract

The combination of oleogelators in oil structuring has an untapped potential, since effective pairs have usually been found by serendipity. The aim of this work was to evaluate the combination of berry (BEW) or sunflower wax (SHW) with glycerol monostearate (GMS) in flaxseed oil (FXO) at 5 and 25 °C. The thermal and mechanical properties, microstructure, and stability of oleogels were investigated. Self-standing and translucent gels were obtained from BEW in FXO. However, the mixture BEW:GMS resulted in a decrease of dynamic moduli. Moreover, changes in the crystal network and a reduction of oil binding capacity were noticed. Thus, the GMS prevented the complete organization of BEW in polyunsaturated chains of FXO. Conversely, a positive interaction was found for GMS:SHW, since both alone were not able to impart the structure in FXO. Interestingly, gel was formed with improved properties, even with a small addition of GMS, although an ideal ratio of 1:1 (GMS50:50SHW) was found. Oxidative stability analysis showed that all gels resembled the behavior of liquid oil (~12.00 meqO_2_/kg) over 30 days storage. Therefore, semi-solid systems with nutritional and techno-functional claims were created by using waxes and fatty-acid derivative oleogelator in a rational fashion; this opened the opportunity to tailor oleogel properties.

## 1. Introduction

Solid fats are commonly used in processed foods to confer technological and sensory properties desired by the consumer [1]. However, trans, saturated, and recently interesterified fats have been associated with negative health related effects, such as increasing the risk of obesity, diabetes, cancer, and cardiovascular diseases [2,3]. In this context, oleogelation alters the physical state of vegetable oils (mainly composed of unsaturated fatty acids), providing a semi-solid characteristic, although without changing their chemical composition [4]. Oleogels are formed by the entrapment of organic solvent (i.e., vegetable oils) within a three-dimensional network, which can be formed by the crystallization of oleogelators [5]. For instance, the mixture of liquid oil and structuring agents is heated to a temperature above the melting point of the oleogelator(s), and during cooling, the crystal network is developed by the establishment of weak interactions, creating a self-standing structure. Polar moieties are stabilized by H-bonding or other polar-polar interactions, while nonpolar moieties occur with induced dipole–dipole interactions (London dispersion force) [6].

A myriad of oleogelators such as fatty acid derivatives, sterols, and polymers have been studied for oleogel formation [7,8,9,10,11]. Among them are natural waxes that are widely used for vegetable oil structuring purposes. Waxes may show different melting, crystallization, and gelation temperatures, as well as a crystal morphology, depending on the chemical composition [12]. Berry wax (BEW) (*Rhus verniciflua)* is mostly composed of short chain fatty acids attached to a glycerol backbone, which confers a low melting point [6]. However, hydrolyzed sunflower wax (SHW) (*Helianthus annuus)* is a high melting point wax that is mainly comprised of fatty acids and fatty alcohols [12]. Glycerol monostearate (GMS) is a monoglyceride comprised of saturated long-chain fatty acid esterified to a glycerol backbone [13].

A relatively high amount of these single oleogelators are usually required for oil structuring. In addition to this concern, the complex hierarchical structure of regular solid fats would would be to mimic, in terms of structural arrangement, a gel system comprised of only one kind of oleogelator. Interestingly, the mixture of different oleogelators can generate positive interactions, which may lead to a decrease in the amount used to provide the desired texture to the semi-solid system [14]. Positive interactions between oleogelators can create interesting soft and complex systems, allowing for the tuning of gel characteristics by properly choosing the components of the mixture that endow a specific solubility balance (governing positive interactions) by changing the ratio of the components. Different molecules may co-assemble in supramolecular gel phases (randomly, specifically, or alternatively) or self-sort as coexisting pure assemblies [15]. Even with such advantages of mixed gels, their investigation still has untapped potential for optimizing the formulations and texture properties.

Thus, we propose a strategy design for a mixed-component oleogel based on the combination of low or a high melting point waxes and a fatty acid derivative as the oleogelator. These systems open up the opportunity to create oleogels with a reduced amount of wax (eventually reducing the waxy mouthfeel) with distinct mechanical and techno-functional properties. Such structures can be incorporated as novel ingredients in food formulations and replace conventional solid fats. Moreover, flaxseed oil (*Linum usitatissimum*) is a valuable alternative to the formation of oleogels due to the health benefits related to the presence of omega-3 fatty acids. Recent studies have shown that this oil has anti-inflammatory properties, and its ingestion might prevent cancer, type II diabetes, and cardiovascular diseases [16,17,18,19]. Therefore, the strategy design of this study was to develop and evaluate semi-solid systems based on the mixture of low and high melting point waxes (BEW and SHW, respectively) combined with glycerol monostearate (GMS) in a highly polyunsaturated vegetable oil (FXO).

## 2. Results and Discussion

### 2.1. Gel Formation

Mono and multicomponent oleogels were prepared with berry wax (BEW), sunflower wax (SHW), and glycerol monostearate (GMS) as oleogelators in flaxseed oil (FXO). Preliminary screening was performed by varying the total oleogelator concentration from 1% to 10% (*w/w*) in order to define the minimum amount (critical gelling concentration) to impart the structure in FXO. Different GMS:BEW and GMS:SHW ratios (100:0, 75:25, 50:50, 25:75, and 0:100) were investigated. After preparation, oleogels were stored at a temperature of 5 or 25 °C, and a tilt test was carried out to analyze oleogel formation (Figure S1).

Temperature exerted an influence on the structuration of the monocomponent GMS100 (Figure S1a–d-1) and multicomponent oleogels of berry wax (GMS50:50BEW and GMS75:25BEW Figure S1a,b-2,3). At the lowest temperature, these gels were self-standing, while at 25 °C they flowed after tube inversion (tilt test). However, multicomponent gels of sunflower wax (Figure S1c,d-2,3,4) and GMS25:75BEW (Figure S1a,b-4) exhibited solid-like properties at both temperatures. Interestingly, the monocomponent oleogel of sunflower wax (GMS0:100SHW Figure S1c,d-5) exhibited more liquid-like characteristics at both temperatures. Therefore, monoglyceride (GMS100) and sunflower wax (GMS0:100SHW) did not form gel in FXO at 25 °C, although a positive interaction occurred with the oleogelators mixture (GMS:SHW). In contrast, BEW was able to structure FXO, but GMS addition induced a negative interaction, weakening or even hindering the development of a 3D network at higher GMS concentrations in the mixture.

GMS has been used as an efficient oleogelator in different oils at 25 °C, such as sunflower oil, high oleic sunflower oil, and coconut oil [20]. However, the lack of gel formation can be associated to the high degree of unsaturation of FXO. The presence of a greater unsaturation degree represents a solvent with a larger molar volume (higher conformational freedom caused by the bended chains and hydrophobicity), which may reflect oleogelator–solvent interactions. For instance, oleogels formed with ethylcellulose showed improved mechanical properties as the oily phase was more unsaturated (canola < soybean < flaxseed oil) [21]. The difference between this work and our results could be at least partly attributed to a different mechanism of gel formation—polymeric strands for ethylcellulose and crystallization from the lamellar phase for GMS. Therefore, the packing of triglycerides affected oil structuring differently, depending on the degree of unsaturation of fatty acid molecules. From these results, further experiments were performed on the systems structured at both 5 and 25 °C (GMS0:100BEW, GMS25:75BEW, GMS25:75SHW, GMS50:50SHW, and GMS75:25SHW).

### 2.2. Isothermal Rheological Measurements

#### 2.2.1. Strain Sweep

**Figure 1:**
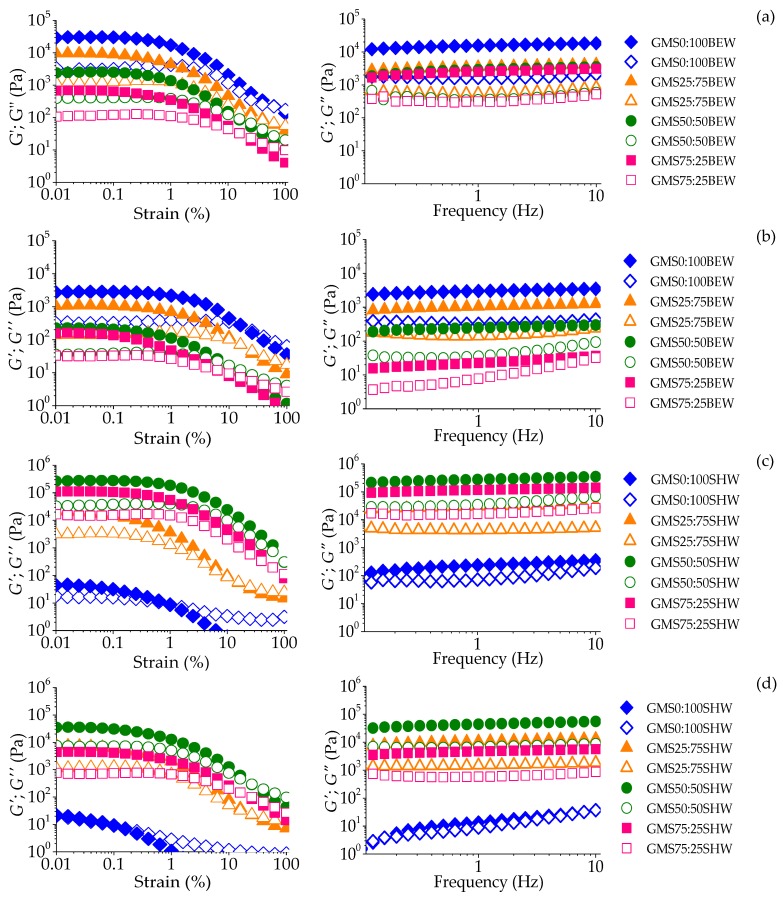
Strain (left) and frequency sweeps (right) for GMS:BEW-based oleogels at 5 °C (**a**), GMS:BEW-based oleogels at 25 °C (**b**), GMS:SHW-based oleogels at 5 °C (**c**), and GMS:SHW-based oleogels at 25 °C (**d**).

Strain sweep measurements were carried out at 5 and 25 °C. Figure 1 shows the plot of strain and frequency sweeps of the mono and multicomponent oleogels. Strain sweep allows for the identification of the linear viscoelastic region (LVR), in which the sample structure does not show irreversible deformation under the application of mechanical forces. The relationship between the applied strain or stress and measured oscillatory stress or strain, respectively, is linear within LVR, and the moduli obtained from the oscillatory response are only a function of the observation time (or frequency) and temperature [22]. Figure 1 shows that *G*′ was higher than *G*′’ within LVR. The maximum limit of LVR was between 0.1% and 1%, although GMS:SHW was more susceptible to the application of mechanical forces. Above these strain values, dynamic moduli showed pronounced decay and a sample response was no longer independent of the magnitude of deformation. At this stage, a crossover point (*G*′ = *G*″) occurred, indicating that gels underwent permanent deformation caused by the rupture of the structural network [23].

BEW monocomponent oleogels (GMS0:100BEW) showed broad LVR and the highest *G*′ values, rather than their mixtures with GMS. A small GMS addition (GMS25:75BEW) did not affect the LVR extension compared to GMS0:100BEW. Interestingly, the length of the LVR of oleogels was slightly longer at 25 °C than at 5 °C, suggesting a greater stability in the strain applied on gels at room temperature. However, these samples showed a stronger network at 5 °C than 25 °C, indicating, by the higher strain values associated with the crossover point, approximately 100% and 10% of the strain, respectively. The increasing addition of fatty acid derivative oleogelator (GMS50:50BEW and GMS75:25BEW) led to a drop in the average value of *G*′ in the LVR (*G’_LVR_*) and smaller strain values associated to the crossover point, disclosing the more fragile nature of these gels. While at 25 °C GMS50:50BEW and GMS75:25BEW a gel-like behavior (*G*′ > *G*″) within LVR was shown, these systems were not self-standing structures (tilt test) (Figure S1).

Unlike gels formed by BEW mixtures, SHW showed a positive effect on the oleogel structure when mixed with GMS (GMS25:75SHW, GMS50:50SHW, and GMS75:25SHW), considering that neither SHW nor GMS formed gels as a single oleogelator at 25 °C (Figure S1). Monocomponent SHW oleogels showed lower average values of *G*′_LVR_, and a narrow length of the linear region compared to the mixtures. Thus, GMS:SHW combination revealed some intriguing rheological outputs, while showing better results than GMS0:100SHW. The higher elastic modulus (*G*′) of the mixtures indicated that the combination of GMS and SHW positively influenced gelation, resulting in improved crystal network formation. After defining the LVR, the samples were subjected to a frequency sweep in order to investigate the observation time dependent behavior of the oleogels.

#### 2.2.2. Frequency Sweep

All the samples, apart from GMS0:100SHW, showed a dominate storage modulus (*G*′, representing the elastic property) over the loss modulus (*G*″, depicting the viscous property), pointing to a gel-like behavior, as can be seen in Figure 1. Moreover, the slight positive slope of the *G*′ data is reported to be observed for soft gels [24]. At 5 °C, the GMS oleogel (GMS100) was shown to be a strong gel with great G’ values (>10^5^ Pa), as presented in Table S1 and Figure S2. However, at 25 °C there was a decrease in the elastic property, which was also confirmed by the visual appearance (Figure S1), which showed precipitation of GMS in the FXO. In this way, at higher temperatures, the crystals were formed, but they were unable to impart a cohesive structure that would lead to oleogelation.

Visual analysis (Figure S1) showed that all BEW-based oleogels were structured at 5 °C, but at 25 °C, only GMS0:100BEW and GMS25:75BEW. These results are in agreement with the values of the dynamic moduli (*G*′ and *G*″), since moduli at 5 °C were higher than at 25 °C. At 5 °C, the samples GMS25:75BEW, GMS50:50BEW, and GMS75:25BEW did not show great differences in dynamic rheological behavior. However, at 25 °C, the increase of the GMS ratio in the mixture of oleogelators caused a decrease in *G*′ values and an increased frequency dependence (Figure 1a,b and Table S1). These results suggest that there was no positive interaction between GMS and BEW as oleogelators, which may have been induced by GMS crystals hindering the complete organization of the BEW crystalline network.

However, GMS0:100SHW did not form visually self-standing systems in FXO at both temperatures. Indeed, SHW was a poor oleogelator in flaxseed oil, which was confirmed by either the low value of elastic component or notable frequency dependence, especially at 25 °C. However, after GMS incorporation, all SHW-based systems showed gel-like behavior and greater values of dynamic moduli. As aforementioned, at 25 °C, neither GMS nor SHW formed oleogel with FXO. Therefore, these single oleogelators were not able to drive intermolecular interactions in FXO that would build a crystalline network at room temperature [25]. This result refutes the hypothesis that the highest degree of unsaturation of the organic solvent can be related to the production of stronger gels [20,26]. This fact suggested that accurate solubility balance must be reached, making it difficult to state that the degree of unsaturation is the determining factor to reach stronger oleogel formation. Interestingly, the reduction of SHW and the addition of GMS led to oleogel formation at 25 and 5 °C, suggesting a good interaction between these structuring agents. However, GMS:SHW ratio of 50:50 showed the strongest oleogel, which was confirmed by the highest *G*′ at 5 and 25 °C.

#### 2.2.3. Thixotropy

Another important property is the capacity of oleogel to recover its viscosity after shearing input. Thixotropy was studied by applying alternate cycles of low and high shear rates (0.1 and 10 s^−1^) to the samples and tracking the changes in viscosity (Table 1) at two different temperatures (5 and 25 °C). Initially samples were subjected to a low shear rate (0.1 s^−1^) and the behavior was similar for all oleogels regardless the studied temperature, showing a decrease of viscosity over time. Such a drop in viscosity, even at relatively low shear rates, suggests a sensitive nature of the gels to shear, which can be related to the weak interactions underpinning the crystal’s 3D network formation (such as van der Waals interactions and London dispersion forces) [27].

The viscosity at the end of this first interval was considered to be the initial viscosity. After that, a remarkable drop in viscosity was observed with the high shear rate step (10 s^−1^). Finally, the samples were subjected again to a low shear rate (0.1 s^−1^) for a further 10 min, in order to observe the structural recovery. The viscosity at the end of this step was considered to be the recovered viscosity, which was used to calculate the relative percentage recovery. In general, the lower temperature favored the initial viscosity, which was in agreement with the higher elastic properties from oscillatory measurements (Table S1). The addition of GMS decreased the initial viscosity of BEW-based oleogel, as presented in Table 1. Conversely, the system GMS:SHW (50:50 ratio) possessed the highest initial viscosity at both temperatures, in comparison to all the formulations.

SHW-based oleogel showed poor regeneration at both 5 and 25 °C, with a maximum of 27.8 ± 0.7%. Such behavior is typical of brittle gels that is in general characterized by a great elastic nature and a narrow LVR [6]. However, from the strain sweep results presented in Figure 1, SHW-based multicomponent oleogels did not show a typical “brittle-type” failure, as they showed a broad yield zone. We suggest that the mixture of SHW and GMS created a heterogeneous network of crystalline particles, promoting a non-uniform bonding strength and a consequent “ductile type” failure [28]. The structure could collapse into smaller clusters of aggregates as gel is sheared, but with the reduction of shearing, the re-establishment of these clusters into a ordered and cohesive network would be hindered, since shear forces overcame the Brownian motion of suspended crystals [6].

Interestingly, BEW-based oleogel showed high viscosity recovery at 25 °C (77.5 ± 2.3%) in FXO, while it presented lower recovery at 5 °C (32.7 ± 0.6%). This result differed from Doan et al. (2015) in which the BEW-based oleogel expressed a higher thixotropic recovery in rice bran oil at 5 °C (84.05%) [29]. The reduction of BEW and the addition of GMS (GMS25:75BEW), however, it can reverse these results, promoting greater restructuring at 5 °C.

### 2.3. Non-Isothermal Measurements

#### 2.3.1. Differential Calorimetry Scanning-DSC

**Figure 2:**
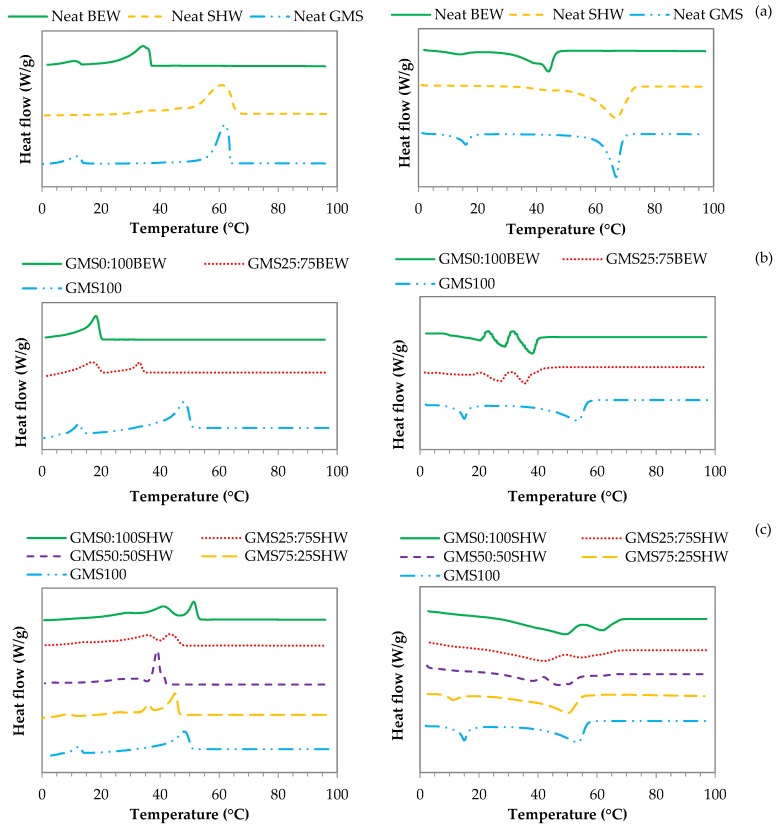
Crystallization (left) and melting (right) for neat BEW, SHW, and GMS (**a**) GMS:BEW-based oleogels (**b**) and GMS:SHW-based oleogels (**c**).

The thermal behavior (onset and peak crystallization and peak melting temperatures) of neat materials (GMS, BEW, and SHW), monocomponents, and mixed oleogels was characterized by DSC, as shown in Figure 2 and Table 2. In Figure 2, the crystallization and melting peak temperatures are depicted, in which BEW presented two well-defined peaks in the cooling step close to 34 °C and 11 °C (Figure 2a). However, three peaks were identified in the second heating step: one small peak at low temperature (~14 °C) and two overlapping peaks at higher temperatures, between 35 and 45 °C (Figure 2a). Pure SHW showed broad crystallization peaks between 25 and 70 °C, and melting peaks ranging from 30 to 75 °C.

The presence of different peaks is related to the multicomponent chemical nature of these waxes. SHW is mainly composed of moieties of fatty alcohols and fatty acids of long-chain (C_20_-C_28_), and a small amount of non-hydrolyzed esters (according to the supplier information). However, BEW primarily consists of free fatty acids with a chain length of C_16_ and C_18_, where most of them are esterified to a glycerol backbone [12]. Pure GMS showed well-defined peaks, with two crystallization sharp peaks in the cooling step (~62 °C and ~12 °C) and two well-defined melting peaks in the second heating cycle (~16 °C and ~67 °C). GMS usually crystallizes in a mixed lamellar structure. The first exothermic peak could correspond to the crystallization of the aliphatic tails and the second one is associated to the polymorphic transition into sub-α structure [30]. As disclosed by the reduction of the second melting enthalpy in Table 2 (from 137.0 ± 0.4 to 100.9 ± 0.8 J/g), during the cooling step, GMS might not return to its more stable crystal-phase (*β*-phase), forming a less dense and ordered crystal packing (α-form) [31].

In turn, the beginning of crystallization process (onset) can provide an extra edge in understanding the thermal behavior of mixed oleogels. The crystallization onset temperatures (*T_C1,onset_*) of oleogels were lower and peaks were less pronounced (lower enthalpy values) than neat waxes (Figure 2 and Table 2) because of the dilution effect. Neat structuring agents were evaluated as the main chemical components in charge of the crystallization, without considering the dispersion in oily phase [23]. The results show that there was an increase in *T_C1,onset_* when GMS was added to the BEW-based oleogel (Table 2). Crystallization of the GMS starts at higher temperatures (around 40 °C), but this process is only consolidated at lower temperature crystallizations (around 20 °C).

The addition of a small amount of GMS (GMS25:75SHW) did not affect the *T_C1, onset_* of the SHW-based oleogels, because both species initialize the crystallization at similar temperatures. However, lower *T_C1,onset_* values were observed at in same proportions of GMS to SHW (GMS50:50SHW). Moreover, a greater difference between ΔHmI and ΔHmII indicates that the crystalline species takes longer to develop (i.e., sequential crystallization), which can be interpreted as a positive interaction of these oleogelators taking place at this specific ratio (1:1) [32]. This positive interaction was corroborated by rheological results, because, in this condition, the gels were stronger. In addition, the thermograms showed that two overlapping peaks were formed, followed by a very sharp peak, probably indicating the concomitant crystallization of distinct species. This suggests that the interaction between these two oleogelators, in this proportion, alters the crystal network structure and can be related to the aforementioned positive interaction, which is capable of forming strong gels. This result differs from that reported by Pérez-Monterroza, Márquez-Cardozo, and Ciro-Velásquez (2014), who evaluated the effect of different ratios of oleogelators on the crystallization temperature of avocado oil [33]. They reported that the decrease in *T_C1, onset_* was related to the negative interaction between beeswax and Span 60, which weakened the oleogel structure. Therefore, finding a combination of gelators is not a simple task and remains a challenge.

Upon combining BEW and GMS or SHW and GMS, a simultaneous crystallization of two different crystal types (co-existence), co-crystallization (one type of mixed crystal), or both can be expected. The formation of some mixed crystals can likely occur because of the interaction between polar moieties of GMS and SHW. Conversely, it seems that GMS indirectly hinders the BEW crystal to form a cohesive network in FXO [32]. When analyzing the relationship between crystallization and gelation for wax-based oleogels, interestingly, *T_sol-gel_* from non-isothermal rheology and the crystallization onset temperature (*T_C1, onset_*) assessed by DSC measurements showed similar values, indicating that these two processes occurred almost simultaneously or an event is a consequence of the other. As reported by Patel et al. (2015), the gelation of low-melting point natural waxes (i.e., berry and fruit wax) is not preceded by the extensive microstructure development, in contrast to the gels formed by waxes containing high and mid-melting components [6].

#### 2.3.2. Temperature Sweep-From Rheological Measurements

**Figure 3:**
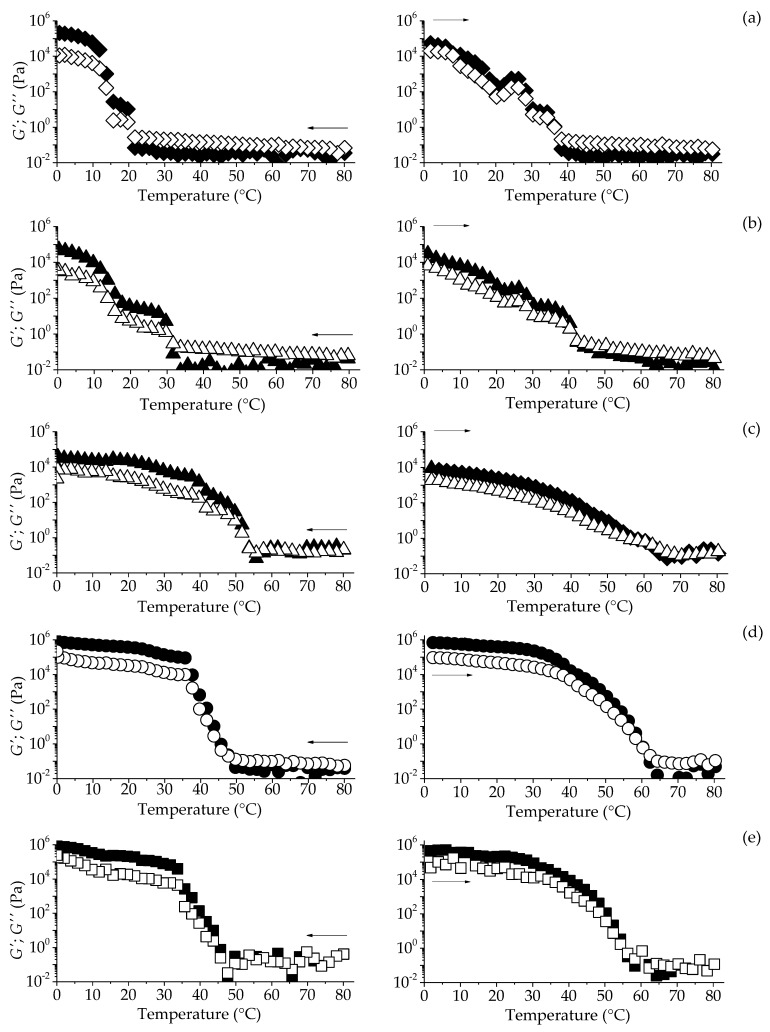
Temperature sweep of GMS0:100BEW (**a**), GMS25:75BEW (**b**), GMS25:75SWH (**c**), GMS50:50SWH (**d**), and GMS75:25SWH (**e**). Cooling (left) and heating steps (right). Solid symbol (*G*′) and open symbol (*G*″).

Non-isothermal rheological measurements were carried out to understand the effect of temperature on the gelling behavior of gels (Figure 3). At the cooling step, *G*″ was first greater than *G*′, indicating a predominance of viscous behavior until the crossover of the dynamic moduli (*G″ = G’*), which was followed by a gradual increase of *G*′ as the temperature reduced. This last period indicates the organization of the crystals until reaching the 3D-network construction, forming the framework of oleogels [34]. All oleogels showed the typical abrupt phase transition to full crystallization, but BEW oleogels showed a slower transition in two steps, around 20 °C (between 30 and 10 °C). Systems with SHW presented an abrupt transition during the cooling step at relatively high temperatures (between 35 and 45 °C for GMS50:50SHW and GMS75:25SHW, and 40–50 °C for GMS25:75SHW), with the subsequent establishment and further strengthening of the crystalline network as the temperature decreased. Furthermore, the GMS50:50SHW exhibited the most pronounced development of viscoelastic properties compared to the other SHW-based oleogels formulations during the crystallization step.

After cooling, the oleogels remained isothermal at 0 °C for a period of time before the subsequent heating step. During heating stage, both monocomponent and mixed BEW-based oleogels showed different steps (slopes), between 20 and 40 °C, indicating the presence of different crystalline species involved in the network formation. Such behavior in GMS0:100BEW can be explained by the multicomponent nature of the waxes and the presence of shorter chain fatty acids (C_16_-C_18_), where most of them are bounded on the glycerol backbones as diacylglycerols (DAGs) and triacylglycerols (TAGs) [32].

Thus, the crystallization of BEW in the oil may have continued during the isothermal period, generating another polymorphic crystalline conformation [23], which was more clearly detected during heating. In contrast, SHW-based oleogels exhibited a constant and gradually decreasing behavior of the dynamic moduli until gel-sol transition *(G″* > *G*′). The gel-sol transition temperatures (*T_gel-sol_* and *T_sol-gel_*) were lower for BEW-based oleogel, athough a small incorporation of GMS in the mixture caused an increase in these values. Nevertheless, multi-component SHW oleogels showed similar *T_gel-sol_* around 60 °C (Table 2). In terms of the technological function, the lower *T_gel-sol_* around 37−42 °C of BEW-based oleogels, compared to SHW oleogels (59−62 °C), can define a different set of applications of both gels.

### 2.4. Microstructure

#### 2.4.1. Polarized Light Microscopy (PLM)

**Figure 4:**
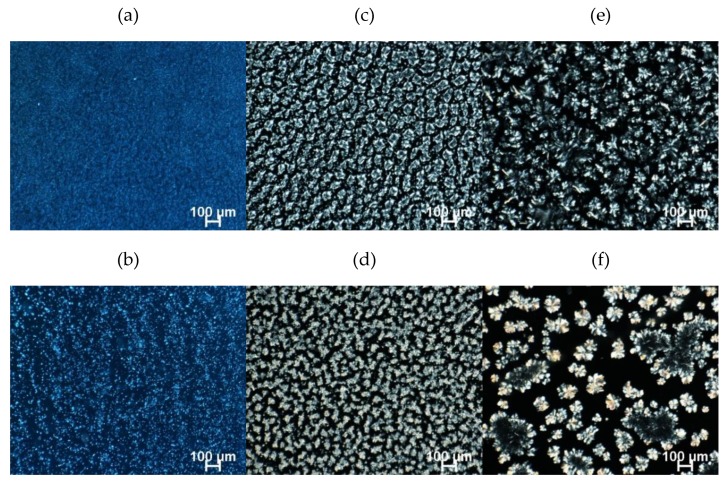
Polarized light microscopy of monocomponent oleogels with total oleogelator concentration of 6% (*w/w*). GMS0:100BEW at 5 °C (**a**); GMS0:100BEW at 25 °C (**b**); GMS0:100SHW at 5 °C (**c**); GMS0:100SHW at 25 °C (**d**); GMS100 at 5 °C (**e**), and GMS100 at 25 °C (**f**).

**Figure 5:**
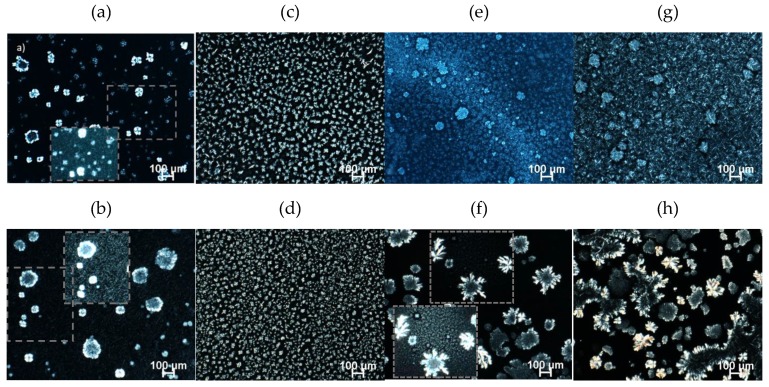
Polarized light microscopy of mixed oleogels with total oleogelator concentration of 6% (*w*/*w*). Top: 5 °C and bottom: 25 °C. GMS25:75BEW (**a**,**b**); GMS25:75SHW (**c**,**d**); GMS50:50SHW (**e**,**f**), and GMS75:25SHW (**g**,**h**).

The micrographs under polarized light were obtained after 48 h of storage at 5 and 25 °C. Figure 4 and Figure 5 present the birefringent crystalline microstructure of the gels, clearly showing that the oleogelator type, ratio of components in the mixture, and storage temperature affected the crystal morphology. After 48 h at 5 °C, the GMS100 crystal network showed rosette-like and spherulite crystals (Figure 4e) that were similar to the oleogel formed by GMS and high oleic sunflower oil [20]. However, bigger GMS clusters precipitated in FXO at 25 °C [35].

The GMS0:100BEW displayed tiny rod-like crystals at 5 °C, forming a dense and cohesive network (Figure 4a) that converged with the translucid visual appearance of this monocomponent oleogel (Figure S1). Similar morphology was found by Doan and To et al. (2017) in rice bran oil [12]. We hypothesized that the shorter chain fatty acids of BEW were able to self organize when embedded in polyunsaturated chains of FXO, leading to oleogel formation with appealing technological characteristics (translucence and good mechanical properties). The crystal size increased and became more dispersed at higher temperature (25 °C) (Figure 4b), but still exhibited a condensed display of crystals. The addition of GMS modified the network morphology of BEW-based oleogels, presenting the combination of spherulitic crystals with tiny needle-like crystals in the background (zoomed area) (Figure 5a). The presence of bigger needle-like crystals and spherulitic crystals in GMS25:75BEW (Figure 5b) was observed at 25 °C. However, these needle-like species are only the edges of the platelet crystals, which can be arise as an experimental artifact from the preparation of the sample on glass slides [36].

Similar to the other monocomponent systems, GMS0:100SHW showed different types of crystals, depending on the storage temperature (Figure 4c,d). However, the self-standing structure could not be formed (6% of oleogelator in FXO) at either 5 or 25 °C. Surprisingly, the combination of GMS and SHW induced oleogel formation and the change in the ratio of components in the mixture (GMS:SHW) clearly affected the crystal morphology. We suggest that the polar heads of GMS interacted with polar moieties of fatty acids and fatty alcohols of SHW, having a positive effect on the crystal structure. The microstructure of GMS25:75SHW system was very similar at both studied temperatures and presented small spherulites crystals, with some branches (Figure 5c,d). However GMS25:75SHW showed a smaller size compared to GMS0:100SHW, which can explain the formation of oleogel.

In the oleogelator mixture containing the same proportion of GMS and SHW, at 5 °C, crystals appear as spherulittes tightly packed with some bigger crystals (Figure 5e). Figure 5f exhibits larger crystals of different shapes (branched dendritic crystals and rosette-like) incorporated within tiny crystalline species (zoom area) for GMS50:50SHW at 25 °C. GMS related crystals, which are the bigger birrefringent structures, coexist with smaller spherulitte crystals.

The further increase of GMS in the mixture (GMS75:25SHW) led to an increase of crystal size. Spherulites crystals can be observed to be radiating and branching outwards within a matrix of small needle-like crystals (Figure 5g) at 5 °C. Whereas Figure 5h reveals larger branched dendritic and rosette-like crystals at 25 °C, ressembling the crystal behaviour of pure GMS since the latter is the prevailing oleogelator in the mixture (Figure 5h).

#### 2.4.2. Scanning Electron Microscopy (SEM)

While PLM can disclose important information about crystal morphology, the electron microscopy technique could provide a better visualization of the crystal network, assuming that the network surface area and surface roughness are the main factors in determining oil structuring ability rather than the morphology of wax crystals [36].

**Figure 6:**
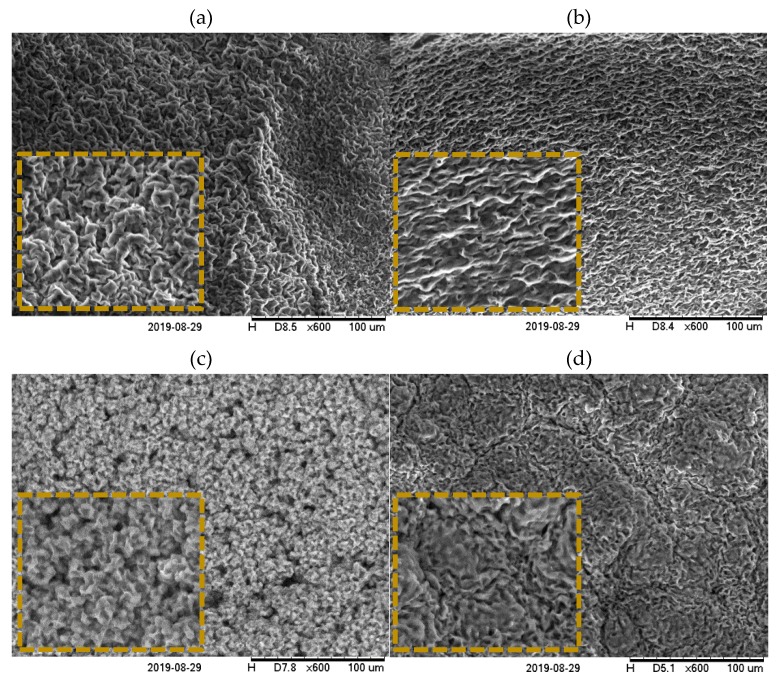
Scanning electronic microscopy (SEM) images of GMS0:100BEW (**a**), GMS25:75BEW (**b**), GMS25:75SHW (**c**), and GMS50:50SHW (**d**) oleogels. Magnification of 600×. Scale equivalent to 100 μm. The inset figures correspond to 1000× magnification.

Some samples were analysed under SEM, revealing more information about oleogels’ microstructure. Figure 6a shows that the GMS0:100BEW oleogel was composed of platelet-like crystals randomly organized. This configuration could be formed from both newly formed nucleation sites or from pre-existing crystals under isothermal conditions [23]. The addition of GMS changed the configuration of the BEW network that appeared as piled platelets upon each other (Figure 6c). However, when dealing with mixtures of high melting point wax (SHW) and monoglyceride, different crystal networks were observed. For instance, GMS25:75SHW (Figure 6c) showed a sponge-like network formed from the aggregation of spherulites, while GMS50:50SHW presented a denser and more compact network ressembling to a platelet organization, but with a different display (Figure 6d) compared to BEW-based gels (Figure 6a).

### 2.5. Oil Binding Capacity (OBC)

The crystalline network is responsible for physical entrapment and immobilization of the organic solvent [37]. The oleogel GMS0:100BEW showed excellent oil holding ability at both 25 and 5 °C (Table 3), with no oil release during accelerated stability tests. This property can be explained by the homogeneity of the dense network formed by small crystals (Figure 4a,b), which reduces the amount of pores and increases the surface area available in order to retain the oil [38]. The weakening of the GMS25:75BEW network is confirmed by the decrease in OBC, which is also aligned with the reduction of elastic properties from rheological measurements (Figure 1a,b). The addition of monoglyceride resulted in the formation of bigger crystals (Figure 5a,b) that hindered the organization of BEW in FXO, facilitating the release of vegetable oil. There was a more pronounced decrease in OBC of these systems at 25 °C, which can be due to the increase of crystal size and weakening of mechanical properties.

The GMS50:50SHW also presented no oil release at 5 °C, however, it did not show statistical difference between the other formulations of SHW. At 25 °C, this oleogel also presented the highest OBC, but with a slight oil release. Microscopy shows that, despite the increase of some crystals in the GMS50:50SHW at this temperature, these crystals were surrounded by a large amount of tiny crystals, which was likely from the wax crystallization (Figure 5e,f). Therefore, it is assumed that these crystals prevent the formation of large voids that would result in further oil release [39]. GMS25:75SHW was the only SHW-based oleogels that showed similar efficiency in oil trapping at both temperatures. According to the microscopy of this sample, there were no major differences in crystal morphology at the two storage temperatures. The GMS75:25SHW exhibited the lowest OBC at 25 °C. This result can be explained by the network consisting of large, different crystals that led to the formation of many gaps and a less structured network, corroborating with rheological measurements (Figure 1c,d).

### 2.6. Oxidative Stability (OS)

**Figure 7:**
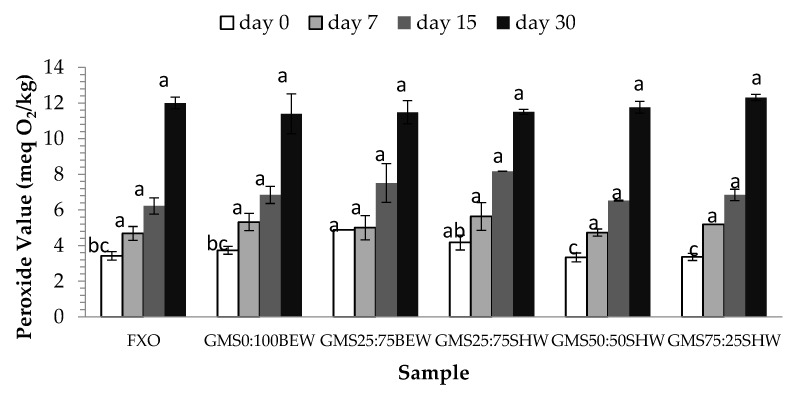
Peroxide value of flaxseed oil and derived oleogels after 0, 7, 15, and 30 day(s) storage at 25 °C. Different letters means statistical difference between all samples for each time.

Oxidation stability of FXO and oleogels was tracked during one month of storage (0, 7, 15, and 30 days) at room temperature (Figure 7). The OS was assessed by peroxide value (PV), which indicates the amount of primary products formed during lipid oxidation [40]. After preparation of oleogels and storage in a refrigeration temperature for 48 h, the first measurement of the PV was carried out (0 day storage). Likewise, neat FXO (without any heating) was used as a control sample and it remained in the same conditions applied for oleogels. For GMS:BEW mixtures at 0 day storage, the reduction of wax content resulted in higher PV. Conversely, the reduction of GMS amount when dealing with the high melting point wax decreased the PV of oleogels. It is noteworthy to mention that, with exception of the control sample (FXO), all oleogels were reheated before each measurement, however, applying a lower temperature than the oleogel preparation temperature (70 °C).

Despite all the heating stages, oleogels showed the same behavior as FXO, even though the oil was not submitted to any thermal stress. While oleogels were subjected to heating during preparation, the samples were quickly transferred to an ice bath just after oleogelator(s) solubilization (heat and agitation). The rapid cooling leads to a faster heat exchange with the air, preventing the increased oxygen solubilization into samples during cooling step [41] and contributed to stability of unsaturated fatty acid chains. However, the three-dimensional network formed was not able to play a physical barrier role in order to protect the organic solvent from light and oxygen effects during storage, which would increase the oleogel stability compared to liquid oil [42]. High peroxide values are usually associated with FXO, which is normally susceptible to more pronounced oxidation due to the high degree of unsaturation [43]. However, all values were within the limit suggested by the Codex Alimentarius (2001) [44] for cold pressed and virgin oils (15 meqO_2_/kg). Interestingly, the oleogelation process (high temperatures applied to solubilize oleogelator(s) apparently did not affect the oxidative stability of oleogels. Such behavior shows the oxidative stability of FXO-based oleogels, disclosing their potential for commercial application in foodstuff.

## 3. Conclusions

Waxes with different chemical composition and melting point temperatures (BEW and SHW) interacted differently with flaxseed oil combined with a fatty-acid derivative oleogelator. BEW chains were able to organize into FXO polyunsaturated (bended) chains forming translucent, low melting point, and stable (with greater OBC values) gels. However, with the reduction of BEW and addition of GMS, the network weakened, indicating that GMS may have hindered the crystals organization of BEW in FXO. However, SHW and GMS were not able to impart the structure in the FXO at 25 °C. However, interestingly, there was a positive interaction between GMS and SHW, which was noted even with the small incorporation of monoglyceride, but with the best GMS:SHW ratio of 1:1. We hypothesized that the polar heads of GMS interacted with the polar moieties of fatty alcohols present in SHW (such alcohols are in lower amount in BEW), increasing the affinity of oleogelators. The use of FXO was determinative in reaching a proper solubility balance, still preserving the crystallization of crystalline species in GMS/SHW mixture, while GMS/BEW mixture was more solubilized. Thus, this study unveiled the importance and contributed in disclosing the possibilities to explore different combinations of oleogelators in a highly unsaturated oil to form systems with tailored textures.

## 4. Materials and Methods

### 4.1. Material

Flaxseed oil (FXO) was kindly donated by Farinhas Integrais Cisbra Ltda (Parambi, Brazil) (composed mainly of 52.28 ± 0.04% *w*/*w* linolenic acid (C18:3), 21.74 ± 0.11% *w/w* oleic acid (C18:1) and 13.79 ± 0.02% *w*/*w* linoleic acid (C18:2)). Sunflower Hydrowax 6607H (SHW, high-melting point: 65−71 °C, mainly comprised of long-chain (C_20_-C_28_) fatty alcohols and fatty acids, and a small amount of non-hydrolyzed esters) and Berry Wax 6290 (BW, low-melting point: 48−54 °C, composed by free fatty acids (C_16_-C_18_) mostly esterified to a glycerol backbone [12]) were kindly provided by from Kahl GmbH & Co, KG (Trittau, Germany). Glycerol monostearate (GMS, comprised of saturated long-chain fatty acid (C_18_) esterified to a glycerol backbone [13,45]) (purity > 95.0%) was purchased from Alfa Aesar (Tewksbury, MA, USA). All other chemical and solvents were of analytical grade and were used without further purification.

### 4.2. Preparation of Oleogels

Monocomponent oleogels from berry wax (GMS0:100BEW), sunflower wax (GMS0:100SHW), and monostearate (GMS100) were prepared as control samples. Likewise, multicomponent formulations of GMS:SHW and GMS:BEW at different ratios (25:75, 50:50, and 75:25) were manufactured to investigate possible positive interactions between these structuring agents. The total oleogelator(s) concentration was fixed at 6% (*w/w*) for all formulations. Oleogels were prepared by heating the oleogelator(s) and FXO mixture at 80 ± 2 °C, under constant stirring (300 rpm) until a clear solution was obtained. After the heating stage, all samples were immediately transferred to an ice bath for 30 min. After cooling, samples were stored at constant temperature (5 ± 1 °C or 25 ± 1 °C) for at least 48 h prior to analyses.

### 4.3. Characterization of oleogels

#### 4.3.1. Rheological Measurements

All rheological measurements were carried out using a stress-controlled rheometer Physica MCR 301 (Anton Paar, Graz, Austria) equipped with a Peltier system. Sandblasted parallel-plate geometry (Ø = 50 mm), with a roughness of 5–7 μm and a gap of 250 μm was used. The isothermal measurements were performed at 5 or 25 °C.

- Strain and frequency sweeps (isothermal measurements)

Strain sweeps at a constant frequency of 1 Hz were performed to determine the linear viscoelastic region (LVR) of oleogels. Then, frequency sweeps (0.01-10 Hz) were carried out with a strain value within the LVR. The frequency-dependent behavior of oleogels was accessed by recording storage (*G*′) and loss (*G”*) moduli as a function of frequency.

- Thixotropy (isothermal measurement)

The capacity of oleogels to recover viscosity after shear was evaluated by means of thixotropic recovery tests. It is an important technological output to foreseen applications of oleogels in food products. First, samples were allowed to rest for 5 min, after that they were subjected to consecutive steps of low (0.1 s^−1^), high (10 s^−1^), and low (0.1 s^−1^) shear rates during 10, 5, and 10 min, respectively. The viscosity recovery percentage of the oleogels was calculated by comparing the final viscosity values after both low shear rate steps [29].

- Temperature sweeps (non-isothermal measurements)

The behavior of the elastic (*G*′) and viscous (*G*″) moduli was investigated during cooling and heating stages, disclosing sol-gel and gel-sol transitions, respectively. Temperature ramps were performed simulating the gel network formation at a rate of 5 °C.min^−1^, with a fixed frequency (1 Hz) and strain value set within LVR. Initially, the equipment was preheated to 80 °C, and then samples were left isothermally for 1 min, cooled from 80 to 0 °C, maintained isothermally for 14 min, and heated again to 80 °C. The crossover temperature (*G*′ = *G*″) during cooling was defined as the gelling temperature (*T_sol-gel_*) and a heating stage was considered as a gel-sol transition temperature (*T_gel-sol_*) [46].

#### 4.3.2. Differential Scanning Calorimetry (DSC)

The thermal properties of oleogels were evaluated using a differential scanning calorimeter, TA Instruments model DSC-2920 (New Castle, USA), with a coupled cooling unit (Refrigerated Cooling Systems). Both a hermetic aluminum pan containing the sample (5–7 mg) and an empty pan used as a reference were placed in the equipment. Samples were initially equilibrated at 0 °C, heated to 100 °C (heating step I), kept in isothermal conditions for 5 min, then cooled to 0 °C (cooling step), kept in isothermal conditions for 30 min, and finally heated to 100 °C again (heating step II). Heating and cooling ramps were performed at a constant rate of 5 °C.min^−1^**.** Data were processed by TA Universal Analysis (TA Instruments, New Castle, Delaware, USA) software defining some properties from thermal curves as the crystallization onset temperature (*T_C1, onset_*) associated with the first crystallization peak and enthalpy corresponding to both the first (ΔHmI) and second (ΔHmII) heating steps [47].

#### 4.3.3. Microstructure

- Polarized light microscopy (PLM)

Crystal morphology of mono and multicomponent oleogels were investigated using a Polarized Light Microscope (Olympus System Microscope model BX 50, Olympus America Inc., Center Valley, USA) equipped with a digital camera (Nikon DS-Ri1, Melville, NY, USA). Images were analyzed with NIS-Elements Microscope Image Software (Nikon, Melville, NY, USA). A small amount of oleogel was placed in a glass slide and gently covered with a cover slip. The following procedure was conducted in order to mimic oleogel preparation conditions: (i) Sample was heated at 80 °C using a hot stage connected to a Linkam T95 System Controller (Linkam Scientific Instrument Ltd., Surrey, UK); (ii) kept in isothermal conditions for 3 min; (iii) cooled to 0 °C at a rate of 5 °C.min^−1^; (iv) maintained in isothermal conditions for additional 14 min, and finally; (v) glass slides were stored for 48 h at 5 °C or 25 °C before images were taken.

- Scanning Electron Microscopy (SEM)

The oleogels were previously deoiled with sequential isopropanol and ethanol washings in order to expose and preserve the crystalline oleogelator network [48]. Then, the total amount of the mixture (oleogel and ethanol) was transferred to a qualitative filter paper (Whatman #1) and ethanol was used to wash the sample while removing the remaining oil. Finally, samples were dried (approximately 48h) to promote solvent evaporation. All sample preparation steps were performed at 5 °C to avoid melting of the crystalline network. The images were taken with an acceleration voltage of 15 kV with a magnification of 600× and 1000× using a scanning electron microscope (TM3000, Hitachi, Japan).

#### 4.3.4. Oil Binding Capacity (OBC)

Oil binding capacity was evaluated performing the methodology described by Blake & Marangoni (2015) with slight modifications [38]. The accelerated stability test was performed by subjecting the oleogel samples to centrifugation at 14,000 rpm (21.8 × 10^3^ g) for 30 min using a microcentrifuge (5418 R Eppendorf, Hamburg, Germany). Approximately 1 g of freshly prepared oleogel was weighed into 1.5 mL tubes. The centrifugation was carried out after oleogels to be stored at 5 or 25 °C during 48 h. Subsequently to centrifugation, the released oil was drained using qualitative filter paper (Whatman #1) by inverting the tubes for 30 min. The percentage of OBC was calculated using Equation (1) [49].

(1) $$\mathrm{OCB}\;\%\;=\;[1-\frac{m_{i}-m_{f}}{m_{i}}]*100$$

where *m_i_* is the weight of the sample before centrifugation and *m_f_* is the weight after the oil drainage.

#### 4.3.5. Oxidative Stability (OS)

OS of liquid oil and oleogels was evaluated by determining the peroxide value (PV) at 0, 7, 15 and 30 days of storage with light and air exposition at room temperature (25 °C) (AOCS, 2003). Previously, oleogels were heated to 70 °C until to be completely melted. An aliquot of 5 g of sample, either unheated flaxseed oil or melted oleogel, was weighed, then, 50 mL of acetic acid-isoctane solution (3:2 *v/v*) and 0.5 mL of saturated potassium iodide solution (144 g of potassium iodide in 100 mL of distilled water) were added. The flask was capped and stirred manually for 1 min, afterwards, 30 mL of distilled water, indicator starch solution (1% *w/v*) and 10 drops of lauryl (100 g of lauryl sulfate in 100 mL of distilled water) were also incorporated into the mixture. Finally, the sample was titrated with 0.01 N sodium thiosulfate solution until the brown color disappeared. The PV value was calculated according to Equation (2):

(2) $$\mathrm{PV}\;(\mathrm{meq}O_{2}/\mathrm{kg})\;=\;\frac{BxNx100}{A},$$

where B is the volume in mL of 0.01 N sodium thiosulphate solution required for titration; N is the normality of the sodium thiosulfate solution; and A is the amount of the sample (g).

#### 4.3.6. Statistical Analysis

The experiments were performed in triplicate and results were presented as mean ± standard deviation. Statistical differences between treatments were evaluated by analysis of variance (ANOVA) and Tukey test (*p* < 0.05) using Statistica software version 7.0 (Statsoft^®^, Tulsa, Oklahoma, USA).

## 5. Patents

A patent application from the study reported in this manuscript has been filed at the patent office.

## Figures and Tables

**Table 1:** Initial viscosity and viscosity recovery percentage in thixotropy measurements.

	Initial Viscosity (Pa.s)		Viscosity Recovery (%)	
Sample	5 °C	25 °C	5 °C	25 °C
GMS0:100BEW	460.5 ± 10.6 ^a;A^	279.7 ± 16.5 ^a;B^	32.7 ± 0.6 ^b;B^	77.5 ± 2.3 ^a;A^
GMS25:75BEW	155.3 ± 27.1 ^b;A^	103 ± 4.2 ^b;A^	56.5 ± 5.0 ^a;A^	26.7 ± 3.7 ^b;B^
GMS25:75SHW	478 ± 33.9 ^c;A^	167 ± 24 ^c;B^	13.1 ± 0.6 ^b;A^	6.1 ± 0.7 ^a;B^
GMS50:50SHW	1465 ± 134.4 ^a;A^	975 ± 34 ^a^	27.8 ± 0.7 ^a;^	11.2 ± 3.1 ^a;B^
GMS75:25SHW	980.5 ± 21.9 ^b;A^	257.7 ± 10.7 ^b;B^	10.4 ± 1.9 ^b;A^	10.5 ±1.5 ^a;A^

Different lower case letters (a, b and c) mean statistical difference between BEW or SHW formulations at 5 or 25 °C. Different capital letters (A and B) mean statistics difference for each sample at both temperatures.

**Table 2:** Crystallization temperature, melting enthalpy from DSC measurements, and temperatures associated to sol-gel and gel-sol transitions from non-isothermal rheological measurements.

Sample	ΔHmI (J/g)	ΔHmII (J/g)	*T_C1, onset_* (°C)	*T_sol-gel_* (°C)	*T_gel-sol_* (°C)
**GMS0:100BEW**	8.51 ± 1.58 ^c;A^	7.91 ± 0.38 ^b;A^	19.83 ± 0.13 ^c^	20.70 ± 0.00 ^b^	37.30 ± 0.00 ^b^
**GMS25:75BEW**	9.73 ± 2.28 ^ab;A^	8.21 ± 0.58 ^b;A^	33.32 ± 1.61 ^b^	30.70 ± 0.00 ^a^	42.30 ± 1.41 ^a^
**GMS100**	18.78 ± 2.96 ^a;A^	16.01 ± 0.83 ^a;A^	50.47 ± 0.35 ^a^	-	-
**GMS0:100SHW**	20.72 ± 0.43 ^a;A^	20.87 ± 1.34 ^a;A^	51.47 ± 2.04 ^a^	-	-
**GMS25:75SHW**	18.88 ± 0.84 ^a;A^	16.58 ± 1.12 ^b;A^	48.90 ± 2.68 ^ab^	53.70 ± 1.41 ^a^	61.97 ± 3.06 ^a^
**GMS50:50SHW**	20.36 ± 2.81 ^a;A^	13.65 ± 0.66 ^b;A^	42.78 ± 1.29 ^c^	45.37 ± 3.06 ^c^	60.30 ± 1.41 ^a^
**GMS75:25SHW**	20.32 ± 0.11 ^a;A^	14.16 ± 0.65 ^b;B^	46.65 ± 0.37 ^ab^	46.70 ± 0.00 ^ab^	59.30 ± 2.00 ^a^
**GMS100**	18.78 ± 2.96 ^a;A^	16.01 ± 0.83 ^b;A^	50.47 ± 0.35 ^a^	-	-
**Neat BEW**	137 ± 0.4	100.8 ± 0.8	37.07 ± 0.15	-	-
**Neat SHW**	209 ± 12	187.8 ± 3.8	65.31 ± 0.26	-	-
**Neat GMS**	137 ± 0.4	100.9 ± 0.8	64.02 ± 0.25	-	-

Different lower case letters mean statistical difference between different BEW or SHW-based formulations. Different capital letters mean statistical differences of enthalpy for each sample in the 1st and 2nd heating cycle.

**Table 3:** Oil binding capacity of mono and multicomponent wax-based oleogels.

	OBC (%)	
Sample	5 °C	25 °C
GMS0:100BEW	100.0 ± 0.0 ^a; A^	96.7 ± 1.2 ^a; B^
GMS25:75BEW	92.7 ± 0.6 ^b; A^	65.1 ± 4.3 ^b; B^
GMS25:75SHW	94.3 ± 6.5 ^a; A^	81.9 ± 6.9 ^b; A^
GMS50:50SHW	100 ± 0.0 ^a; A^	93.4 ± 0.7 ^a; B^
GMS75:25SHW	99.7 ± 0.2 ^a; A^	58.2 ± 2.4 ^c; B^

Different lower case letters mean statistical difference between BEW or SHW formulations at 5 or 25 °C. Different capital letters mean statistical differences for each sample between temperatures.

## Supplementary Materials

The following are available online at https://www.mdpi.com/2310-2861/6/1/5/s1, Figure S1: Visual appearance of oleogels from berry wax (BEW), sunflower wax (SHW), glycerol monostearate (GMS), GMS:BEW (a, b), and GMS:SHW (c, d) in flaxseed oil (FXO) after from 48 h between 5 °C (a, c) and 25 °C (b, d). GMS:BEW and GMS:SHW ratios of 100:0 (1); 25:75 (2); 50:50 (3); 75:25 (4); and 0:100 (5). Figure S2: Frequency sweep for the control sample GMS100. Solid symbol (*G*′) and open symbol (*G*″). Square symbols are related to data collect at 5 °C and circle symbol at 25 °C. Table S1: Mean and standard deviation of dynamic moduli at 1 Hz frequency.
