# Supplementary material for: Tailoring Properties of Mixed-Component Oleogels: Wax and Monoglyceride Interactions Towards Flaxseed Oil Structuring
Source: Gels. 2020 Jan 31;6(1):5. doi: 10.3390/gels6010005 (PMC7151330; doi:10.3390/gels6010005)
Supplement: Supplementary file 1 [file gels-06-00005-s001.pdf]

Supplementary Materials:

Table S1. Mean and standard deviation of dynamic moduli at 1 Hz frequency.

Oleogel	*G′* (Pa)	*G″*(Pa)
	**5 °C**	
**GMS0:100BEW**	15700 ± 1385^{b}	1623 ± 122^{b}
**GMS25:75BEW**	3513 ± 697^{c}	552 ± 55^{c}
**GMS50:50BEW**	2910 ± 612^{c}	355 ± 75^{c}
**GMS75:25BEW**	2487 ± 414^{c}	316 ± 108^{c}
**GMS100**	306000 ± 9899^{a}	26100 ± 707^{a}
**GMS0:100SHW**	237 ± 55 ^{c}	72 ± 7^{c}
**GMS25:75SHW**	29600 ± 1198^{c}	4270 ± 1924^{c}
**GMS50:50SHW**	275333 ± 39272^{a}	34633 ± 5781^{a}
**GMS75:25SHW**	116000 ± 12124^{b}	16200 ± 1249^{b}
**GMS100**	306000 ± 9899^{a}	26100 ± 707^{a}
	**25 °C**	
**GMS0:100BEW**	3006 ± 162^{a}	323 ± 8^{a}
**GMS25:75BEW**	1050 ± 26^{b}	139 ± 4^{b}
**GMS50:50BEW**	249 ± 42^{cd}	35 ± 4^{d}
**GMS75:25BEW**	22 ± 5^{d}	7 ± 1^{e}
**GMS100**	511 ± 21^{c}	68 ± 10^{c}
**GMS0:100SHW**	13 ± 5^{d}	8 ± 1^{c}
**GMS25:75SHW**	10826 ± 2312^{b}	1506 ± 356^{b}
**GMS50:50SHW**	44200 ± 282^{a}	6765 ± 7^{a}
**GMS75:25SHW**	4715 ± 289^{c}	588 ± 51^{c}
**GMS100**	511 ± 21^{cd}	68 ± 10^{c}

Different lower case letters mean statistical difference between BEW or SHW formulations at 5 or 25 °C. Different capital letters mean statistical difference for each sample between temperatures.

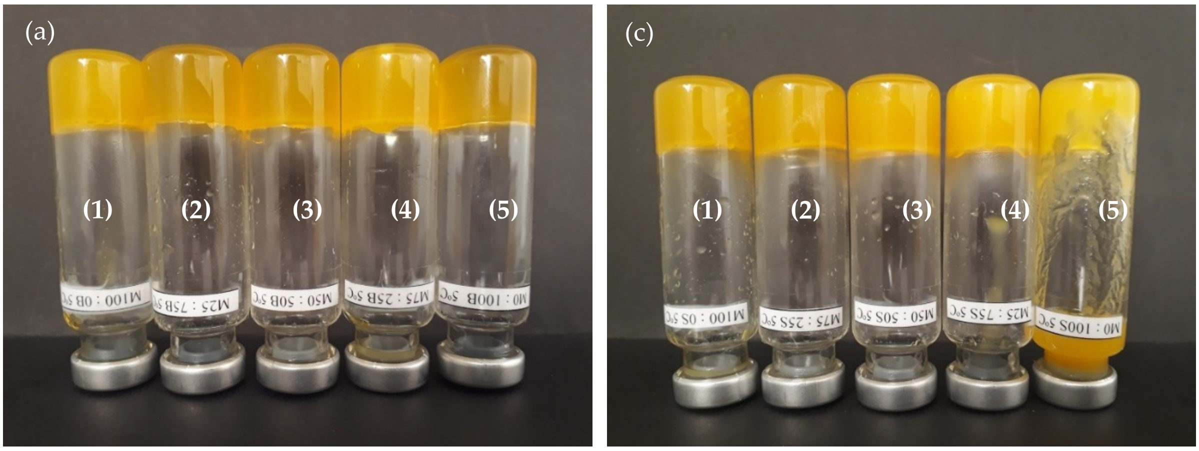

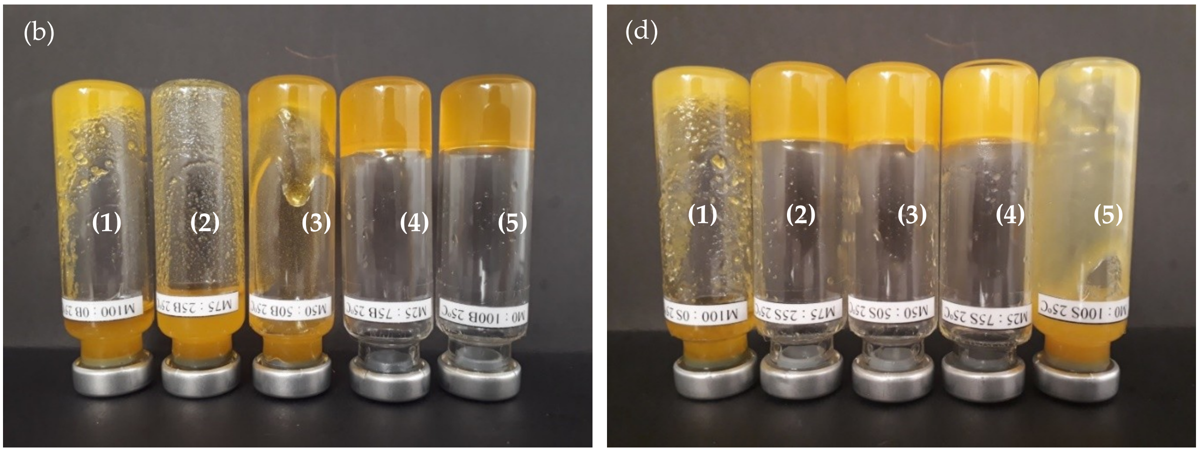

Figure S1. Visual appearance of oleogels from berry wax (BEW), sunflower wax (SHW), glycerol monostearate (GMS), GMS:BEW (a, b), and GMS:SHW (c, d) in flaxseed oil (FXO) after from 48 h between 5 °C (**a**, **c**) and 25 °C (**b**, **d**). GMS:BEW and GMS:SHW ratios of 100:0 (1); 25:75 (2); 50:50 (3); 75:25 (4); and 0:100 (5).

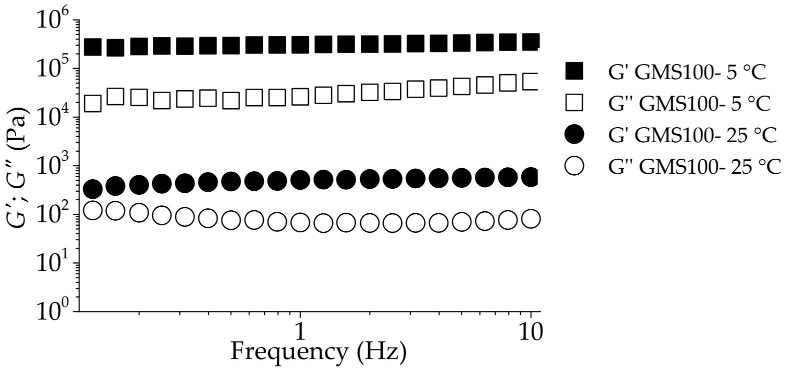

Figure S2. Frequency sweep for the control sample GMS100. Solid symbol (*G′*) and open symbol (*G′′*). Square symbols are related to data collect at 5 °C and circle symbol at 25 °C.
